# Adjuvanted Vaccine Induces Functional Antibodies against *Pseudomonas aeruginosa* Filamentous Bacteriophages

**DOI:** 10.3390/vaccines12020115

**Published:** 2024-01-24

**Authors:** Valery C. Román-Cruz, Shannon M. Miller, Roman A. Schoener, Chase Lukasiewicz, Amelia K. Schmidt, Blair L. DeBuysscher, David Burkhart, Patrick R. Secor, Jay T. Evans

**Affiliations:** 1 Division of Biological Sciences, University of Montana, Missoula, MT 59812, USA; valery.roman-cruz@umconnect.umt.edu (V.C.R.-C.); amelia.schmidt@mso.umt.edu (A.K.S.); patrick.secor@mso.umt.edu (P.R.S.); 2 Center for Translational Medicine, University of Montana, Missoula, MT 59812, USA; chaseluka@gmail.com (C.L.); blair.debuysscher@mso.umt.edu (B.L.D.); david.burkhart@mso.umt.edu (D.B.); 3 Inimmune Corporation, Missoula, MT 59802, USA; shannon.m.miller@inimmune.com (S.M.M.); roman.a.schoener@inimmune.com (R.A.S.); 4 Department of Biomedical & Pharmaceutical Sciences, University of Montana, Missoula, MT 59812, USA

**Keywords:** *Pseudomonas aeruginosa*, Pf bacteriophage, adjuvant, TLR4 agonist, vaccine

## Abstract

*Pseudomonas aeruginosa* (*Pa*), a WHO priority 1 pathogen, resulted in approximately 559,000 deaths globally in 2019. *Pa* has a multitude of host-immune evasion strategies that enhance *Pa* virulence. Most clinical isolates of *Pa* are infected by a phage called Pf that has the ability to misdirect the host-immune response and provide structural integrity to biofilms. Previous studies demonstrate that vaccination against the coat protein (CoaB) of Pf4 virions can assist in the clearance of *Pa* from the dorsal wound model in mice. Here, a consensus peptide was derived from CoaB and conjugated to cross-reacting material 197 (CRM197). This conjugate was adjuvanted with a novel synthetic Toll-like receptor agonist (TLR) 4 agonist, INI-2002, and used to vaccinate mice. Mice vaccinated with CoaB-CRM conjugate and INI-2002 developed high anti-CoaB peptide-specific IgG antibody titers. Direct binding of the peptide-specific antibodies to whole-phage virus particles was demonstrated by ELISA. Furthermore, a functional assay demonstrated that antibodies generated from vaccinated mice disrupted the replicative cycle of Pf phages. The use of an adjuvanted phage vaccine targeting *Pa* is an innovative vaccine strategy with the potential to become a new tool targeting multi-drug-resistant *Pa* infections in high-risk populations.

## 1. Introduction

*Pseudomonas aeruginosa* (*Pa*) is a Gram-negative bacterium that often infects diabetic ulcers, burn wounds, implanted medical devices, and the lungs of cystic fibrosis patients [1]. *Pa* is typically treated with antibiotics, but emerging multi-drug-resistant strains are problematic. Multi-drug-resistant *Pa* was placed on the 2018 global priority one list by the World Health Organization [2,3]. A surveillance study identified 12% of isolates tested in European intensive care units as multi-drug resistant, and in 2019, a global multidrug-resistant surveillance study found that of 13 million bacterial infection-related deaths, approximately 4% were related to *Pa* infections [4,5].

The search for an effective vaccine against *Pa* has been ongoing for half a century. However, only three vaccine candidates reached phase III clinical trials, and each ultimately failed primary efficacy endpoints [6]. Most previous vaccine candidates have used *Pa* surface proteins or other bacterial-expressed proteins as vaccine targets. Unfortunately, *Pa*’s ability to rapidly adapt or modulate the expression of target antigens has prolonged the arduous journey of finding an effective vaccine [6]. In addition to the ability to evade vaccine-mediated host defenses, *Pa* has an arsenal of strategies to evade natural host immunity, leading to serious and life-threatening chronic infections [7]. One such mechanism is *Pa’s* ability to develop biofilms, which can lead to a reduction in surveillance by the adaptive immune system and antibiotic efficacy [8]. 

Most strains of *Pa* are infected by a prophage called Pf integrated into the chromosome as a prophage [9]. *Pa* strain PAO1 is infected by the Pf4 prophage, which has been identified as a virulence factor and provides structural integrity to *Pa* biofilms [9]. Pf4 phage belongs to the Inoviridae family and is structurally a long, filamentous virion. The majority of the Pf4 virion is composed of repeated copies of the major coat protein, CoaB, packaging a circular single-stranded DNA genome [9]. The Pf4 is lysogenic; it can passively replicate as a prophage without lysing its bacterial host. External signals received by the *Pa* host can result in the excision of Pf4 prophage, leading to the production of infectious virions [10]. At sites of infection, Pf4 virions are internalized by immune cells via endocytosis, where they activate Toll-Like receptor (TLR) 3 signaling, leading to the production of type I interferons while also reducing TNF-α production [7]. This misdirected antiviral immune response to a bacterial pathogen reduces antibacterial immune responses, such as reduced phagocytic uptake by macrophages [7]. 

Because Pf4 virions are a key virulence factor of *Pa* and Pf prophages are present in most *Pa* strains, Pf virions could be a viable vaccine target. CoaB is highly conserved, with previous studies identifying a conserved portion of the coat protein that spans 699 isolates of *Pa* [7]. The N-terminus of this conserved portion is largely exposed to the environment and is predominantly comprised of negatively charged amino acids [11]. Due to its highly conserved nature, a portion from this region was used to develop a 19-amino acid consensus peptide, thus creating a simple and novel vaccine antigen covering the majority of the *Pa* clinical isolates [7]. Previous murine vaccination studies demonstrated that a humoral immune response against the Pf4 phage results in protection against *Pa* and clearance of infection in a murine wound infection model [7]. 

Here we optimized the peptide-carrier conjugate vaccine, containing the 19 amino acid CoaB consensus peptide, through carefully controlled conjugation to CRM197 using a heterobifunctional crosslinker and the addition of synthetic adjuvants to enhance immunogenicity. An adjuvant is an additive in a vaccine that improves vaccine efficacy, resulting in longer-lasting, more robust protection [12]. Some next-generation adjuvants have the ability to target pattern recognition receptors (PRR) on immune cells. Adjuvants bound to PRRs can result in the production of specific cytokines and cell-surface proteins, which can enhance adaptive immunity when co-administered with an antigen [12]. The lead adjuvant described herein, INI-2002, is a synthetic TLR4 agonist that enhances both cell-mediated and humoral immunity [13]. Animals vaccinated with the CoaB peptide conjugated to CRM197 and adjuvanted with INI-2002 had increased antigen-specific antibody production when compared to animals vaccinated with the CoaB conjugate adsorbed to Alhydrogel. Furthermore, the INI-2002 adjuvanted vaccine induced functional antibodies that bound directly to Pf4 phages and disrupted the phage replicative cycle. Creating a safe and effective vaccine that can mount a robust immune response against Pf4 phage is an important advancement towards the development and clinical testing of phage-based vaccines targeting multi-drug-resistant *Pa* infections.

## 2. Materials and Methods

### 2.1. INI-2002 Compound Synthesis

INI-2002 was synthesized using a linear approach as previously described [13,14].

### 2.2. CoaB Conjugate to CRM

In order to conjugate CRM197 (provided by Inimmune Corporation, Missoula, MT, USA) to CoaB (peptide sequence GVIDTSAVESAITDGQGDMC, MW 1968, >95% purity) (Boc Sciences, New York, NY, USA), the heterobifunctional crosslinker, N-γ-maleimidobutyryl-oxysuccinimide ester (GMBS; Thermo Scientific, Waltham, MA, USA), was dissolved in DMSO (Sigma-Aldrich, St. Louis, MO, USA) at a concentration of 25 mg/mL. GMBS reacts with the primary amine functional group of Lysine on CRM197. To achieve 16–17 copies of GMBS in CRM197, 85 equivalents of GMBS solution were added to CRM197 and subsequently allowed to incubate in a water bath for 30 min at 25 °C. Unreacted GMBS was removed by ultrafiltration using 30 kDa Amicon (Millipore, Burlington, MA, USA) Ultra centrifugal filters and buffer, exchanging the intermediate repeatedly with fresh 0.2 M Sodium phosphate buffer (pH = 7.2). MALDITOF MS (Bruker, Billerica, MA, USA) was used to determine the number of GMBS molecules added to one molecule of CRM197; this was confirmed by an observed change in mass. The GMBS-activated CRM197 results in a specific reaction between the attached GMBS and the Cysteine from the CoaB peptide via a thioether. The GMBS-activated CRM197, which was at a concentration of 1.5 mg/mL, was directly added to 40 molar equivalents of peptide and incubated for two hours at 25 °C. The remaining unattached peptide was removed by ultrafiltration using 30 kDa Amicon Ultra centrifugal filters and washed repeatedly with 10 mM Phosphate Buffer pH = 7.2. The peptide-CRM conjugate was sterile filtered using a 0.22 µm PVDF syringe filter (Millex, Duluth, GA, USA). MALDI-TOF MS was used to determine the peptide copy number.

### 2.3. Bacterial Strains, Plasmids, and Growth Conditions

Strains, plasmids, and their sources are listed in Table 1. Unless indicated otherwise, bacteria were grown in lysogeny broth (LB) with gentamicin (10 µg/mL^−1^) at 37 °C with shaking. To induce the expression of plasmid-encoded proteins, a final concentration of 0.1% arabinose was used. 

### 2.4. P. aeruginosa and Pf4 Propagation

*P. aeruginosa* strain PAO1 was plated onto an LB agar plate and incubated at 37 °C for 18 h. The following day, an isolated colony was used to inoculate 500 mL of LB broth, then incubated at 37 °C and 250 rpm until the cultures reached an optical density (OD_600_) of 0.3. The cultures were then infected with Pf4 virions at a multiplicity of infection (MOI) of 10 virions:1 bacterium and incubated for 18 h at 37 °C. The culture was centrifuged at 16,000× *g* for 1 min to remove cells, and the supernatant was filtered through a sterile 0.22 µm syringe filter. The Pf4 virion titer was determined by plaque-forming units (PFU) using *P. aeruginosa* PAO1∆Pf4 as an indicator strain. Aliquots of 50 µL of the Pf4 lysate were stored at −20 °C [17].

### 2.5. In Vivo Experiments

Animal studies were carried out in accordance with the University of Montana’s IACUC guidelines for the care and use of laboratory animals. For murine vaccine studies, groups of 6–10 female C57Bl/6 mice were vaccinated intramuscularly (right gastrocnemius) on days 0 and 14 with the indicated dose of INI-2002 (TLR4 adjuvant) with or without Alhydrogel (InvivoGen, San Diego, CA, USA) and CoaB-CRM conjugate in a volume of 50 µL per injection. Compounds and antigen were diluted as needed in 2.5% glycine in 1X PBS. At 14 days following each vaccination, blood samples were collected via submandibular vein (d14) or cardiac puncture (d28). For evaluation of cell-mediated immune responses, spleens were harvested from a subset of mice on day 19 (5 days post-boost vaccination). For some experiments, a third booster vaccination was administered on day 28, and blood was collected on day 42 for evaluation of humoral immunity. 

### 2.6. Pf4 Serum Inhibition Assay

*P. aeruginosa* ∆Pf4GmR [17] was grown in LB at 37 °C and 250 rpm to an OD_600_ of 0.2. Pf4 virions were diluted in biological triplicate in 20 μL of sera from unvaccinated animals, sera from vaccinated animals, or 1X PBS to obtain a MOI of 0.01 and incubated at 37 °C for 1 h. Pf4 virions co-cultured with sera or 1X PBS were added to *Pa* culture (*Pa* culture was grown to an OD_600_ of 0.3). Timepoints were collected directly prior to the addition of Pf4 +/− sera and 5 min, 15 min, 45 min, 2 h, 4 h, and 16 h after the addition. At each time point, 250 μL of culture was removed and spun at 16,000× *g* for two minutes. The supernatant was transferred to a fresh tube and frozen at −20 °C. Supernatants were serially diluted 1:10 in 1X PBS and spotted in technical triplicate on the lawns of MPAO1 ∆Pf4GmR. Plaque-forming units (PFUs) were counted following 18 h incubation at 37 °C and analyzed using the unpaired Student’s *t*-test (GraphPad Prism version 5.0, San Diego, CA, USA). *p* values of <0.05 were considered statistically significant. 

### 2.7. ELISA for Anti-CoaB Peptide Antibody Quantification

Blood was collected from mice 14 days post-vaccination, and serum was isolated and diluted according to the expected antigen-specific antibody titers (between 1:20 and 1:500). Nunc MaxiSorp plates were coated with 100 µL of CoaB peptide (GVIDTSAVESAITDGQGDM) at 10 µg/mL overnight at room temperature. Plates were washed with 1X PBS plus 0.05% tween 20 and then blocked with 200 µL of SuperBlock (Scytek Laboratories, Logan, UT, USA) for 2 h at 37 °C. Next, serially diluted serum at a dilution factor of 1:3 was added and incubated at 37 °C for 2 h, then washed three times with 1X PBS plus 0.05% tween-20. Following the washing step, plates were incubated with anti-mouse IgG, IgG1, IgG2b, or IgG2c-HRP secondary antibody at a dilution of 1:1000 (Southern Biotech, Birmingham, AL, USA) for 1 h at 37 °C and then washed the same as above. Plates were then incubated with 100 µL of TMB substrate (BD Biosciences, Franklin Lakes, NJ, USA) at room temperature for an hour, followed by absorbance being read at 650 nm using the Spectra Max 190 (Molecular Devices, San Jose, CA, USA). Antibody titers were determined using the XL fit software program version 5.5.05 (IDBS, Boston, MA, USA) at an OD of 0.3. Negative values were assigned a point value of 0.5 in order to plot points and follow up with statistical analysis. 

### 2.8. Phage ELISA

Blood was collected from mice 14 days post-vaccination; serum was isolated and diluted according to the expected antigen-specific antibody titers (between 1:20 and 1:500). Nunc MaxiSorp plates were coated overnight (20 °C) with 100 ul/well of whole phage virions collected from *P. aeruginosa* strain PAO1 at a concentration of 2 × 10^9^ PFU/mL. Plates were then washed with KPL wash buffer (SeraCare, Milford, MA, USA) and blocked with 200 μL of blocking agent made up of 1% BSA and 0.1% goat serum in 1X PBS for one hour at 37 °C. Plates were then incubated with serially diluted serum at a dilution factor of 1:3 for 2 h at 37 °C. Plates were then washed (1X PBS plus 0.05% tween 20) and incubated with anti-mouse IgG, IgG1, or IgG2c-HRP secondary antibody at a dilution of 1:1000 (Southern Biotech, Birmingham, AL, USA) for 1 h at 37 °C. After another wash, 100 µL of TMB substrate (BD Biosciences, Franklin Lakes, NJ, USA) was added, and plates were read at 650 nm using the Spectra Max 190 (Molecular Devices, San Jose, CA, USA). Antibody titers were determined by using the XL fit software program at an OD of 0.3. 

### 2.9. Cell-Mediated Immunity Analysis

Spleens were harvested from five mice per group five days post-booster vaccination (d19). Cells were processed by mechanical disruption of the spleens through a 100 µm filter (Foxx Life Sciences, Salem, NH, USA). Red blood cells were lysed by incubation with red blood cell lysis buffer (Sigma-Aldrich, St. Louis, MO, USA) for 5 min, followed by washing in 1X PBS. Cells were plated in a sterile 96-well polystyrene flat bottom plate at 5 × 10^6^ cells/well in 200 µL of complete Roswell Park Memorial Institute (RPMI) 1640 media (10% heat-inactivated fetal bovine serum, 0.1% 2-Mercaptoethanol, and 1% Penicillin-streptomycin-L-Glutamine). For secreted cytokines, splenocyte single cell suspensions were plated at 5 × 10^6^ in 96-well plates and incubated with 5 µg/mL of CRM or CoaB peptide for 72 h at 37 °C and 5% CO_2_. Supernatants were harvested, and cytokine levels were measured by the MesoScale Discovery (MSD, Rockville, MD, USA) U-PLEX Assay Platform to detect mouse IFNγ, IL-17, TNFα, IL-2, IL-10, and IL-5 following the manufacturer’s instructions.

## 3. Results

### 3.1. Production and Characterization of INI-2002 Adjuvanted CoaB Peptide-CRM Conjugate Vaccine

**Figure 1:**
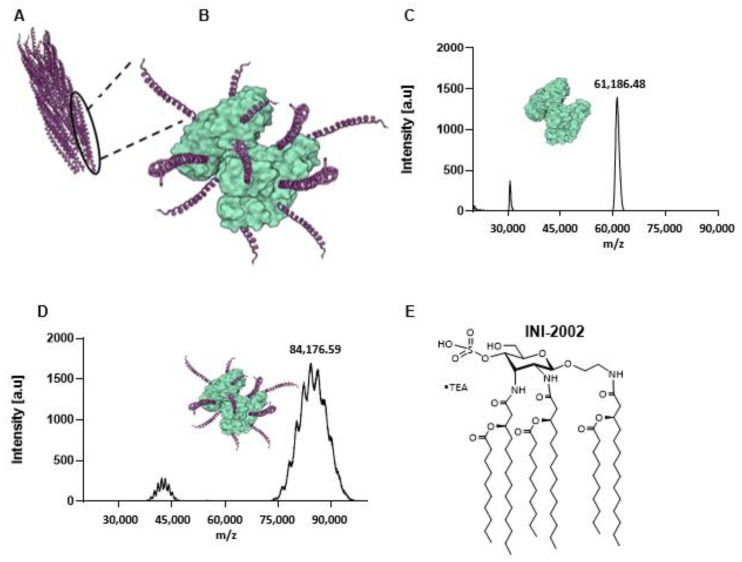
Components of the Pf Phage vaccine and characterization of the CRM-CoaB conjugate. (**A**) Schematic of Pf4 phage crystal structure (PDB: 6TUP); (**B**) Schematic of CoaB peptide GVIDTSAVESAITDGQGDMC conjugated to CRM197 (PDB: 4AE0); (**C**) MALDI-TOF characterization of GMBS-activated CRM197; (**D**) MALDI-TOF characterization of CRM-CoaB conjugate; (**E**) Structure of TLR4 agonist, INI-2002.

For the antigen component of our vaccine, we identified a consensus peptide sequence derived from the coat protein (CoaB) of phages isolated from 669 *P. aeruginosa* strains [11] (Figure 1A). Cross-reacting material 197 (CRM) has been approved by the FDA as a carrier protein in several hapten-conjugate vaccines approved for use in humans [18,19]. CRM is a non-toxic carrier protein-derived from a diphtheria toxin [20] and was selected to assist in amplifying the immunogenicity of the CoaB peptide by conjugation (Figure 1B). The conjugate vaccine CoaB-CRM was prepared by covalently linking the 20 amino acid CoaB peptide (19 aa CoaB peptide with C-terminal cysteine added for conjugation) to the carrier protein CRM. This was accomplished using the heterobifunctional crosslinker GMBS (N-γ-maleimidobutyryl-oxysuccinimide ester). The production of the vaccine can be summarized in two major steps. Firstly, the activation of the protein with GMBS, renders it reactive toward the cysteine residue contained in the peptide sequence. Secondly, the conjugation of the peptide motif to the activated CRM protein. The addition of GMBS to CRM results in a peak shift, indicating the activation of CRM. The level of CRM activation was determined through MALDI-TOF MS (peak at approximately 60,000), the minor peak at approximately 30,000 represents m/2 (double charged product) (Figure 1C). The activated CRM was then incubated with CoaB peptide. The cysteine on the peptide reacted with the activated CRM, following the mechanism of a Michael addition. Incubation was followed by repeated ultrafiltration to remove unreacted peptides. The CoaB copy number (defined as the number of attached CoaB peptides per CRM molecule) was determined through MALDI-TOF (Figure 1D). Throughout the following experiments, a copy number of approximately 10–11 was used.

To further enhance the immunogenicity of the vaccine, we selected a synthetic TLR4 agonist as the adjuvant. Previous studies identified the importance of lipopolysaccharide (LPS) engagement of TLR4 in the clearance of *Pa* infections [21], and thus we hypothesized that the use of a TLR4 agonist would enhance an antibody response specific to CoaB. A component of LPS, Lipid A, is the bioactive element and is the structural basis of our synthetic TLR4 agonist, INI-2002 (Figure 1E) [13,22].

### 3.2. INI-2002 Adjuvanted CoaB-CRM Vaccine-Enhanced CoaB-Specific Antibody Titers

**Figure 2:**
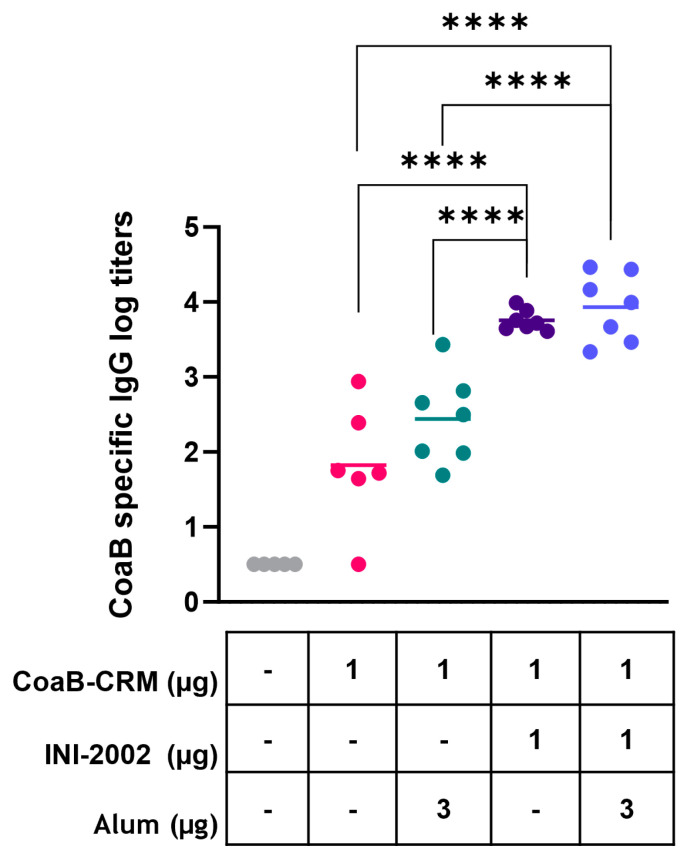
INI-2002 adjuvanted CoaB-CRM results in higher CoaB-specific titers compared to alum adjuvanted CoaB-CRM. C57BL/6 mice (6 or 7 per group) were injected once on days 0 and 14 i.m. with 1 µg/mouse. CRM-CoaB conjugate vaccine adjuvanted with 1 µg/mouse INI-2002 and/or with the indicated dose of alum. Serum was collected on day 14 post-secondary vaccination. CoaB peptide-specific IgG antibody titers were measured by ELISA. The symbols above individual points denote statistical significance. Pink circles represent individual mouse CoaB specific antibody titers from CoaB-CRM group, green circles are CoaB-CRM + alum, purple circles are CoaB-CRM + INI-2002, blue circles are CoaB-CRM + INI-2002 + alum. Line indicates the mean titer for each group. Ordinary one-way ANOVA followed by Fisher’s LSD was used to determine statistical analysis, where **** *p* ≤ 0.0001.

We first determined whether the addition of the INI-2002 adjuvant increased anti-CoaB serum IgG antibody titers over those of CoaB-CRM alone or CoaB-CRM adjuvanted with Alhydrogel. We hypothesized that using two adjuvants together, INI-2002 and Alhydrogel, would further increase anti-CoaB antibody production. To test this hypothesis, C57BL/6 mice were vaccinated intramuscularly (IM) with 1 µg of CoaB-CRM alone, in combination with INI-2002, with Alhydrogel, or with both adjuvants on days 0 and 14. On day 28, sera was collected, and CoaB peptide-specific IgG titers were measured by ELISA. Animals vaccinated with CoaB-CRM in combination with INI-2002 had significantly (*p* < 0.0001) increased CoaB peptide-specific serum IgG titers when compared to all other groups (Figure 2). Surprisingly, IgG titers from animals vaccinated with CoaB-CRM in combination with both INI-2002 and Alhydrogel were not significantly higher than animals vaccinated with CoaB-CRM in combination with INI-2002 (Figure 2). We therefore selected INI-2002 as the lead adjuvant for further CoaB-CRM vaccination and characterization studies.

### 3.3. INI-2002 Enhances Humoral Immunity to CoaB Peptide in a Dose-Dependent Fashion

**Figure 3:**
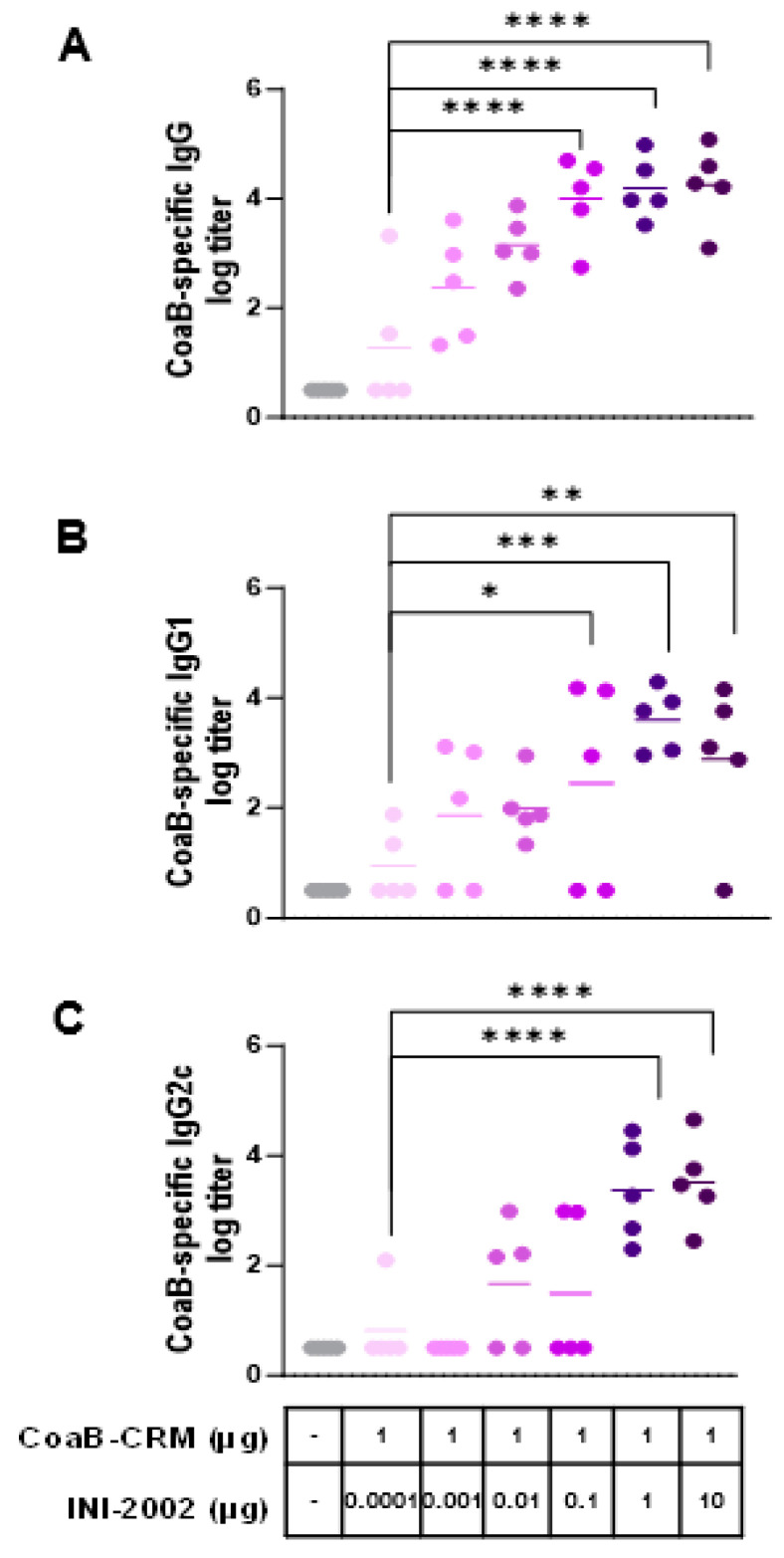
Anti-CoaB IgG, IgG1, and IgG2c antibody titers increase in a dose-dependent manner. C57 Bl/6 mice (5 per group) were injected once on days 0 and 14 i.m. with 1 µg/mouse CRM-CoaB conjugate vaccine adjuvanted with INI2002 as indicated. Serum was collected on day 14 post-secondary vaccination. CoaB peptide-specific IgG (**A**), IgG1 (**B**), and IgG2c (**C**) antibody titers were measured by ELISA. Circles represent individual mouse CoaB specific antibody titers and the shade of purple indicates increasing dose of INI-2002 from 0.0001 to 10 ug. Line indicates the mean titer for each group. The symbols above individual points denote statistical significance. Ordinary one-way ANOVA followed by Fisher’s LSD were used to determine statistical analysis, where * *p* ≤ 0.05, ** *p* ≤ 0.01, *** *p* ≤ 0.001, and **** *p* ≤ 0.0001.

Next, we optimized the dose of INI-2002 as an adjuvant for CoaB-CRM. We hypothesized that increasing the dose of INI-2002 would lead to an increase in anti-CoaB peptide antibody titers in a dose-dependent manner. Mice were vaccinated IM on days 0 and 14 with 1 µg CoaB-CRM and increasing doses of INI-2002 ranging from 0.0001 to 10 µg, as noted in Figure 3. Serum was harvested on day 28 (14 days following the secondary vaccination), and anti-CoaB peptide IgG titers were determined by ELISA. Anti-CoaB IgG titers increased in an INI-2002 dose-dependent manner, with titers plateauing at a dose of 0.1 µg INI-2002 (Figure 3A). Next, we evaluated both CoaB-specific IgG1 and IgG2c antibody titers as an indication of class switching and Th1/2 polarization of the T helper cell response [23]. Anti-CoaB IgG1 and IgG2c titers generally mirrored those of IgG, with the peak titers generated with a 1 µg INI-2002 dose. Mice vaccinated with 1 µg of CoaB-CRM and 1 µg of INI-2002 had significantly (*p* = 0.0006) higher IgG, IgG1, and IgG2 antibody titers when compared to naïve or CoaB-CRM + 0.0001 µg of adjuvant (which exhibited little to no measurable adjuvant activity) (Figure 3B,C). The strong increases in both antigen-specific IgG1 and IgG2c suggest a mixed Th1/2 T helper response to the conjugate.

### 3.4. INI-2002 Adjuvant Enhances Humoral Immunity across the Antigen Dose Range and Is Antigen-Dose Sparing

**Figure 4:**
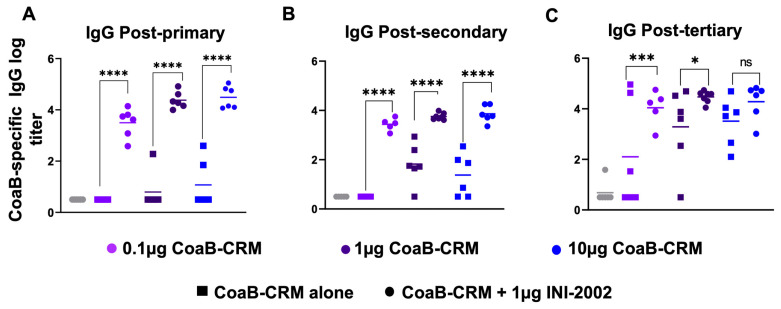
INI-2002 is antigen dose-sparing and enhances CoaB-specific antibodies across a wide range of CoaB-CRM doses. C57Bl/6 mice (10 or 15 per group) were vaccinated on day 0, day 14, and day 28 i.m. with CRM-CoaB conjugate as indicated with or without 1 µg INI-2002. CoaB peptide-specific IgG antibody titers were measured from serum collected on days 14 post-primary (**A**), 14 post-secondary (**B**), and day 14 post-tertiary (**C**). Circles and squares represent individual mouse CoaB specific antibody titers. Squares denote CoaB-CRM alone, circles denote CoaB-CRM plus INI-2002. Colors represent increasing dose of CoaB-CRM. Line indicates the mean titer for each group. The symbols above individual points denote statistical significance. Ordinary one-way ANOVA followed by Fisher’s LSD were used to determine statistical analysis where * *p* ≤ 0.05, *** *p* ≤ 0.001, and **** *p* ≤ 0.0001.

To determine if INI-2002 has an antigen dose-sparing effect and identify the optimal dose of CoaB-CRM, mice were vaccinated with 0.1 µg, 1.0 µg, or 10 µg of CoaB-CRM with or without 1 µg of INI-2002. Serum was collected at 14 days following the primary, secondary, or tertiary vaccination to assess anti-CoaB antibody titers. Mice that received INI-2002 in combination with 0.1, 1, or 10 µg of CoaB-CRM exhibited a statistically significant (*p* < 0.0001) increase in anti-CoaB IgG antibody titers following a primary or secondary vaccination compared to dose-matched mice that received antigen alone (Figure 4A,B). After three vaccinations, the addition of INI-2002 to CoaB-CRM results in a statistically significant boost when 0.1 or 1 µg of CoaB-CRM is used when compared to unadjuvanted dose-matched controls. However, the increase measured with 10 µg of CoaB-CRM plus 1 µg of INI-2002, following three vaccinations, did not reach significance (Figure 4C) when compared to the respective unadjuvanted group, suggesting a plateau in antibody response and corresponding loss of adjuvant impact. The 0.1 µg dose of CoaB-CRM plus INI-2002 resulted in significantly (*p* < 0.0001 post-primary, *p* < 0.0001 post-secondary) higher serum antibody titers compared to the 10 μg dose of CoaB-CRM (no adjuvant) post-primary and post-secondary but not post-tertiary. These data demonstrate at least a 100-fold antigen dose-sparing effect with the addition of the INI-2002 adjuvant. Since 1 µg of CoaB-CRM in combination with 1 µg of INI-2002 resulted in significantly increased CoaB-specific antibody titers following a primary, secondary, or tertiary vaccination compared to antigen alone, this dose was selected as the lead vaccine formulation for further development.

### 3.5. INI-2002 Induces a Balanced T-Helper Cell Response with CRM

**Figure 5:**
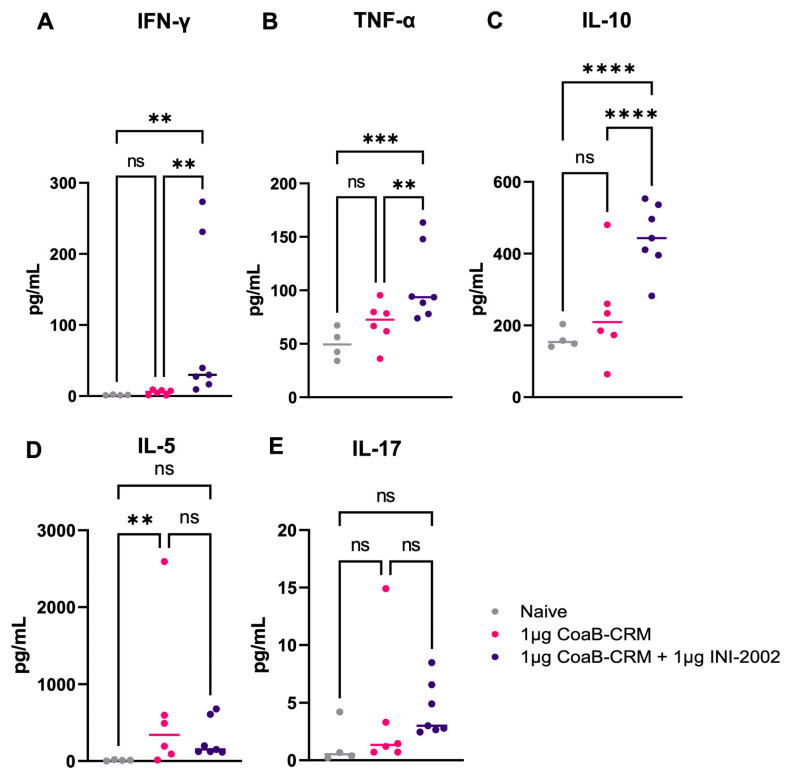
INI-2002, in combination with the CoaB-CRM antigen, promotes cell-mediated immunity to CoaB-CRM. 5 days post-secondary vaccination, mice were euthanized, and spleens were harvested, disaggregated, and restimulated with 5 µg/mL of CoaB-CRM. The following cytokines: IFNγ (**A**), TNFα (**B**), IL-10 (**C**), IL-5 (**D**), and IL-17 (**E**) were measured by an MSD cytokine array. Grey circles represent cytokine levels from individual naïve mice, pink circles are from CoaB-CRM vaccinated mice, purple circles are CoaB-CRM + INI-2002 vaccinated mice. Line indicates the mean titer for each group. Ordinary one-way ANOVA followed by Fisher’s LSD were used to determine statistical analysis, where ** *p* ≤ 0.01, *** *p* ≤ 0.001, and **** *p* ≤ 0.0001.

As noted above, the balanced anti-CoaB-specific IgG1 and IgG2c responses suggested the lead vaccine combination induced a well-balanced T cell response to the associated conjugate. To explore the effects of INI-2002 on T cell help, the cell-mediated immune responses to the carrier protein (CRM197) and peptide were assessed. Mice were vaccinated IM with 1 µg of CoaB-CRM and INI-2002 or CoaB-CRM alone on days 0 and 14. Spleens were harvested 5 days following the secondary vaccination, mechanically disaggregated, and re-stimulated with 5 µg/mL of CoaB-CRM or 5 ug/mL of the CoaB peptide for 72 h. Culture supernatants were harvested and evaluated for secreted cytokines using a multiplex cytokine assay (Meso Scale Discovery). Because the antigen includes a well-studied vaccine carrier protein (CRM197), we anticipated a robust cell-mediated immune response to the carrier and minimal cell-mediated responses to the peptide. Upon re-stimulation of splenocytes from vaccinated mice with the peptide alone, no increase in secreted cytokine was detected (Supplemental Figure S1). Splenocytes from animals vaccinated with 1 µg of CoaB-CRM plus 1 µg of INI-2002 and re-stimulated ex vivo, with CRM exhibited an increased Th1 immune response, as demonstrated by increased IFNγ and TNFα cytokine production compared to their antigen alone and naïve counterpart (Figure 5A,B). Significantly increased IL-10 was also noted in antigen-plus adjuvant-vaccinated animals, suggesting a balanced multi-functional T-cell response (Figure 5C). These data, along with the IgG1 and IgG2c antibody titers (Figure 3), indicate that INI-2002 drives a balanced adaptive immune response when co-administered with CoaB-CRM.

### 3.6. Antibodies Generated from CoaB-CRM-Vaccinated Animals Recognize Native Pf4 Virions

**Figure 6:**
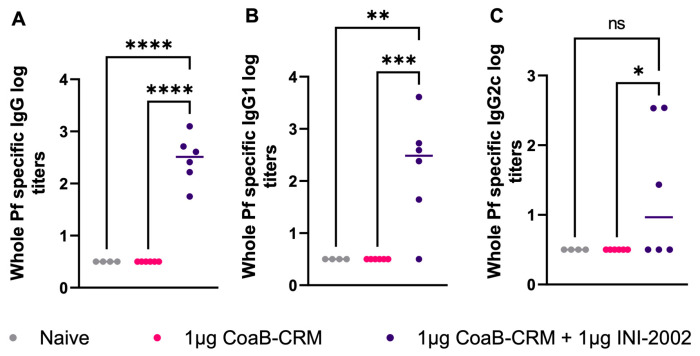
Vaccination with CoaB-CRM + INI-2002 produces whole-phage-specific antibodies. Mice were vaccinated, IM, with 1 µg CoaB-CRM and 1 µg INI-2002 or 1 µg of CoaB-CRM alone on days 0 and 14. Mice were bled on day 28, and serum antibody titers against whole Pf virions were measured by ELISA. Whole-phage-specific (**A**) IgG, (**B**) IgG1, and (**C**) IgG2c antibody titers were measured. Grey circles represent anti-phage titers from individual naïve mice, pink circles are from CoaB-CRM vaccinated mice, purple circles are CoaB-CRM + INI-2002 vaccinated mice. Line indicates the mean titer for each group The symbols above individual points denote statistical significance. Ordinary one-way ANOVA followed by Fisher’s LSD were used to determine statistical analysis, where * *p* ≤ 0.05, ** *p* ≤ 0.01, *** *p* ≤ 0.001, and **** *p* ≤ 0.0001.

The above data demonstrate that serum antibodies generated by vaccination of mice with CoaB-CRM or CoaB-CRM and INI-2002 bind to the CoaB peptide. Next, we evaluated if the anti-CoaB peptide antibodies were capable of binding to whole Pf4 virions. *P. aeruginosa* strain PAO1 was used to generate and purify Pf4 virions as described in the Materials and Methods. Pf4 virions were coated on ELISA plates, and Pf4-specific IgG, IgG1, and IgG2c titers were measured by ELISA. Serum from mice vaccinated with the lead vaccine candidate had significantly higher Pf4-specific IgG (*p* < 0.0001) (Figure 6A), IgG1 (*p* < 0.0005) (Figure 6B), and IgG2c (*p* < 0.036) (Figure 6C) antibody titers compared to mice vaccinated with CoaB-CRM alone or naïve mice. These data demonstrate that antibodies generated from CoaB peptide-vaccinated animals recognize and bind the Pf4 virions. Interestingly, while CoaB-CRM (no adjuvant) induced CoaB peptide-specific antibodies (see Figure 2 and Figure 4), these antibodies did not measurably bind Pf4 virions, suggesting that the INI-2002 adjuvant altered both the titer and specificity of the polyclonal antibody response.

### 3.7. Anti-Pf4 Antibodies Disrupt the Pf4 Replication Cycle

**Figure 7:**
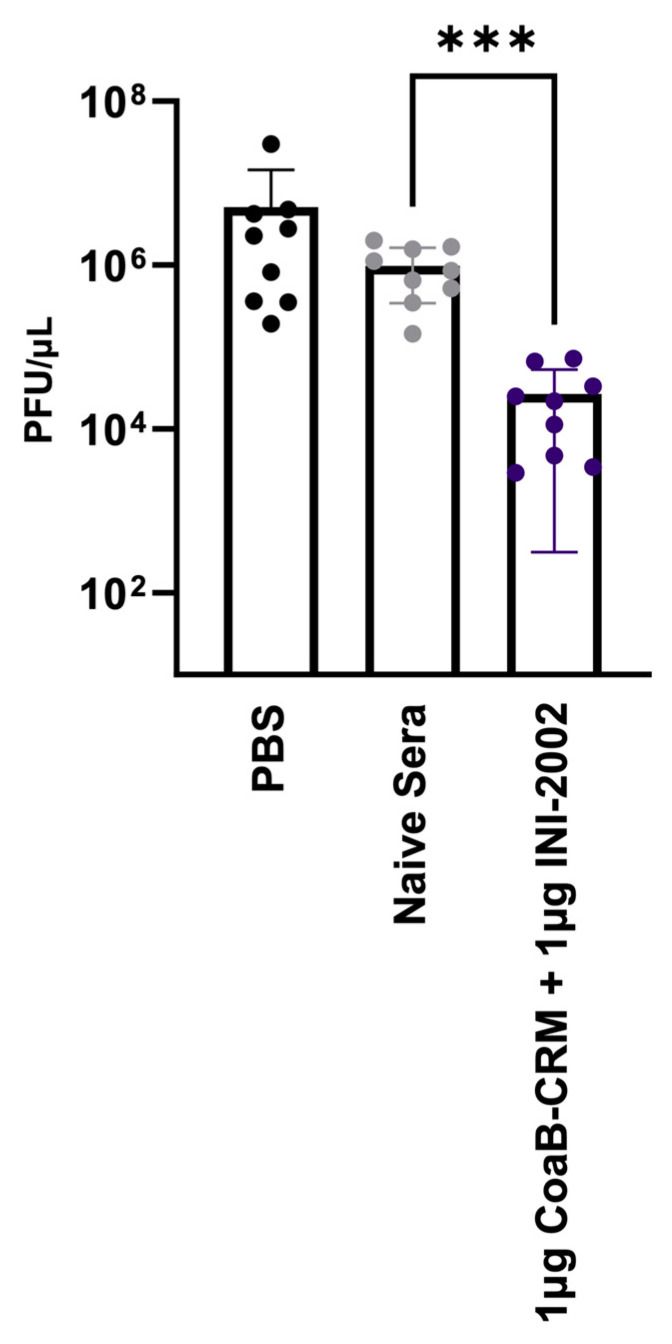
CoaB-specific antibodies disrupt the Pf replication cycle. Pf phage was incubated with either PBS, sera from naïve animals (naive serum), or sera from vaccinated animals (CoaB-CRM + INI-2002) and then spotted on the lawn of ΔPf:Pao1 for the determination of PFUs. Each data point represents an average (PFU/mL) from technical triplicates, and all data points collectively represent three separate experimental repeats. The symbols above individual points denote statistical significance. Ordinary one-way ANOVA followed by Fisher’s LSD were used to determine statistical analysis where *** *p* ≤ 0.001.

Sweere et al. previously demonstrated that CoaB peptide-specific antibodies can protect mice from *P. aeruginosa* challenge [7]. To further these important findings, we sought to explore a possible mechanism for this protection. We tested whether the antibodies present in serum from CoaB-CRM + INI-2002-vaccinated mice could disrupt the Pf4 replicative cycle. Serum from vaccinated mice, naive mouse serum, or 1X PBS was co-cultured with Pf4 virions for 1 h. These cultures were then added to a culture of ∆Pf4GmR (strain lacking the Pf4 prophage) at an OD_600_ of 0.3 to achieve an MOI of 1 virion per 1 × 10^4^ bacterial cells and incubated for 18 h. The supernatant was collected and spotted on the lawns of PAO1 and ∆Pf4. The successful replication of Pf4 phages can be enumerated by the formation of plaques on of PAO1 ∆Pf4 lawns. Plaques were counted, and plaque-forming units per mL (PFU/mL) were calculated. Cultures incubated with serum from animals vaccinated with INI-2002 adjuvated CoaB-CRM showed a significant (*p* < 0.001) reduction in PFU/µL compared to cultures incubated with naive mouse serum (Figure 7). These data indicate that antibodies generated in mice immunized with CoaB-CRM + INI-2002 produced functional antibodies against Pf4, preventing antibody-bound virions from infecting *Pa* and disrupting the Pf4 phage replicative cycle.

## 4. Discussion

The search for a vaccine against *Pa* infections has been ongoing for over 50 years, with only three vaccine candidates reaching phase III clinical trials. One such vaccine candidate was Aerugen^®^, a vaccine comprised of Toxin A from *Pa* conjugated to *O*-polysaccharides. Intranasal administration of Aerugen^®^ showed protection in murine burn wound and lung infection models, but unfortunately, in clinical trials, there was no significant difference between individuals treated with Aerugen^®^ and those receiving the placebo [24,25]. One other vaccine that showed promise was a bivalent vaccine-derived from *Pa* flagellum. Unfortunately, this vaccine showed only 34% protection in acute infections and 55% protection in chronic infections during a phase III clinical trial [25]. *Pa* proteins offer an attractive vaccine target, but ultimately the challenge of these potential targets lies in *Pa*’s ability to reduce expression of certain proteins or mutations that arise in *Pa* proteins to avoid detection by the adaptive immune system [6]. In addition, *Pa* evades the host immune system through the use of biofilms to sequester *Pa* from immune surveillance. As an alternative vaccine strategy, we employed the use of a CoaB peptide-derived from a consensus sequence of the coat protein from Pf4 phage. This phage infects about 60% of *Pa* strains, enhances *Pa* virulence, contributes to biofilm formation, and increases *Pa* chronicity [9]. The development of an adjuvanted vaccine enhancing the humoral immunity to Pf4 phages could provide an alternative vaccine approach against *Pa* infections. The addition of a novel synthetic TLR4 agonist adjuvant, INI-2002, results in a balanced immune response, significantly higher humoral immunity, and disruption of the Pf4 replication cycle [23,24].

Another major finding reported here is that incorporating the INI-2002 adjuvant allows for antigen dose sparing without a reduction in antibody titer. With the incorporation of INI-2002, seroconversion is achieved after a primary injection at all antigen concentrations tested, whereas when vaccinated with CoaB-CRM antigen alone, seroconversion was only achieved after multiple vaccinations with 10–100 times higher antigen doses. Reducing the antigen dose per vaccine means increasing the quantity of available vaccines during high demand. Additionally, reducing antigen concentration has been associated with increased affinity for antibodies and a lower likelihood of T-cell exhaustion post-vaccination compared to higher doses of antigen [26,27]. Dose-sparing vaccine strategies, such as the incorporation of INI-2002, can enhance protection while also limiting costs and improving vaccine efficacy. The efficient binding of antibodies from the CoaB-CRM plus INI-2002 group to whole Pf4 virions and the lack of measurable binding from CoaB-CRM control serum suggest an increased affinity and/or breadth of polyclonal responses with the addition of INI-2002 adjuvant.

Here, we showed that the INI-2002 adjuvanted CoaB-CRM vaccination resulted in a balanced CoaB-specific adaptive immune response. Our collective data showed that when mice were vaccinated with 1 µg of CoaB-CRM and 1 μg of INI-2002, there was a balanced IgG1/IgG2c antibody production specific for the CoaB peptide and native phage. Additionally, vaccinated animals had a significant increase in TNF-α production (a pro-inflammatory cytokine), which has been implicated in enhanced clearance of *P. aeruginosa* in the lung [28]. Furthermore, IL-10 (an anti-inflammatory cytokine), which was also significantly increased in vaccinated animals, has been implicated in moderating inflammation and reducing tissue damage in *Pseudomonas aeruginosa* lung infections [29]. This balanced immune response produced by the vaccine may lead to a reduction in tissue damage while simultaneously enhancing an appropriate inflammatory response.

The use of a peptide-based vaccine is not an entirely novel concept for *Pa*. Previous pre-clinical studies have used peptide-based vaccines derived from *Pa* proteins such as elastase and flagellar proteins to generate high antibody titers [30,31]. Where our vaccine differs from previous studies is the targeting of a Pf4 bacteriophage, a highly conserved virulence factor for *Pa*. Sweere et al. previously demonstrated that a humoral immune response against the Pf4 phage results in protection against *Pa* and clearance of infection in a murine wound infection model [7]. A monoclonal antibody targeting CoaB demonstrated increased protection in direct comparison to the CoaB-KLH + alum adjuvanted vaccine, indicating that an optimized vaccine could have additional benefits. We have extended the previous findings by Sweere et al. using a well-characterized carrier protein (CRM197), a carefully optimized, reproducible, and scalable heterobifunctional crosslinker, complete analytical characterization of the conjugate vaccine, and the selection of an adjuvant to enhance immunogenicity. Optimizing the dose of CoaB-CRM conjugate and including a synthetic TLR4-based adjuvant, INI-2002, increased humoral immunity, promoted IgG2c antibody responses, and significantly increased binding of antibodies to Pf4 phages. These antibodies that bound Pf4 phages also had the functional capability to disrupt the replicative cycle of Pf4. We hypothesize that Pf4 phage-specific antibodies bind to the highly conserved and exposed negatively charged surface domain of Pf4 and reduce the efficiency with which Pf4 can enter and infect a *Pa* host. In vivo, this could result in reduced biofilm integrity, enhanced recognition and clearance of *Pa* harboring Pf4, and give the host a chance to clear the infection before a biofilm can be established.

Although animals dosed with our lead vaccine produced high phage-specific antibody titers with the ability to disrupt the Pf4 phage replicative cycle, our study has some limitations, such as the use of murine models. Although mice can give us valuable data regarding how the vaccine can influence the immune system, the ability to translate the data to the human immune response is limited. Additionally, CoaB epitope sequence homology data across a broad range of *Pa* clinical isolates suggests that targeting 2–4 clades of Pf may be necessary to improve the breadth of immunity in future studies [32].

For future work, we wish to further characterize the immune-stimulatory properties of our peptide-based vaccine. We will determine if the CoaB-CRM antigen in combination with INI-2002 will confer protection in a *Pa* challenge in both small and large animal models. As previous studies have shown, antibodies generated against the Pf4 phage are important in the clearance of *Pa* from the host [7]. The antibody-mediated clearance can be attributed to multiple effector functions of antibodies, which can be addressed in future studies. Specifically, identifying which antibody subtypes have the ability to generate the best protection against *Pa* infection could guide efforts to further optimize a vaccine for enhanced breath and durability through the expansion of protective antibody subtypes.

As previous efforts to develop an effective vaccine against *Pa* have failed, novel approaches are required that look beyond traditional approaches to antigen targets. Our data demonstrates that CoaB-CRM in combination with INI-2002 generates high levels of phage-specific functional antibodies, which have the potential to generate protection against a *Pa* challenge. Previous studies have suggested engaging TLR4 enhances *Pa* bacterial clearance [21], thus the use of INI-2002 may have additional benefits beyond increased antigen-specific antibody titers in a *Pa* infection setting. Preliminary data suggests Pf4-specific antibodies can accelerate and promote clearance of *Pa*-infected wounds [7], thus enhancing Pf4-specific antibodies through the use of INI-2002 as an adjuvant will likely increase cure rates or may prevent *Pa* colonization and biofilm formation. Thus, the creation of a vaccine that incorporates, for the first time, a phage-based antigen in combination with a novel synthetic TLR4 agonist opens the door to vaccine approaches against multi-drug-resistant *Pseudomonas aeruginosa* infections.

## Figures and Tables

**Table 1:** Bacterial strains, phages, and plasmids used in this study.

Strain	Description	Source
*P. aeruginosa*		
PAO1	Wild type	[15]
PAO1 ∆Pf4*GmR*	Pf4 prophage deletion, replaced with GmR cassette. Att sites were not retained.	[15,16]

## Data Availability

Research data and summaries are available by contacting Dr. Jay Evans (jay.evans@mso.umt.edu).

## Supplementary Materials

The following supporting information can be downloaded at: https://www.mdpi.com/article/10.3390/vaccines12020115/s1, Figure S1. INI-2002 in combination with CoaB-CRM antigen promotes cell-mediated immunity to CRM.
