# Supplementary material for: Adjuvanted Vaccine Induces Functional Antibodies against Pseudomonas aeruginosa Filamentous Bacteriophages
Source: Vaccines (Basel). 2024 Jan 24;12(2):115. doi: 10.3390/vaccines12020115 (PMC10892371; doi:10.3390/vaccines12020115)
Supplement: Supplementary file 1 [file vaccines-12-00115-s001.zip › vaccines-2724296-supplementary.pdf]

Article

Adjuvanted vaccine induces functional antibodies against *Pseudomonas aeruginosa* filamentous bacteriophages

Valery C. Román-Cruz, Shannon M. Miller, Roman A. Schoener, Chase Lukasiewicz, Amelia Schmidt, Blair DeBuysscher, David Burkhart, Patrick R. Secor, Jay T. Evans

Supplemental Information:

Supplemental Figure S1. INI-2002 in combination with CoaB-CRM antigen promotes cell mediated immunity to CRM.

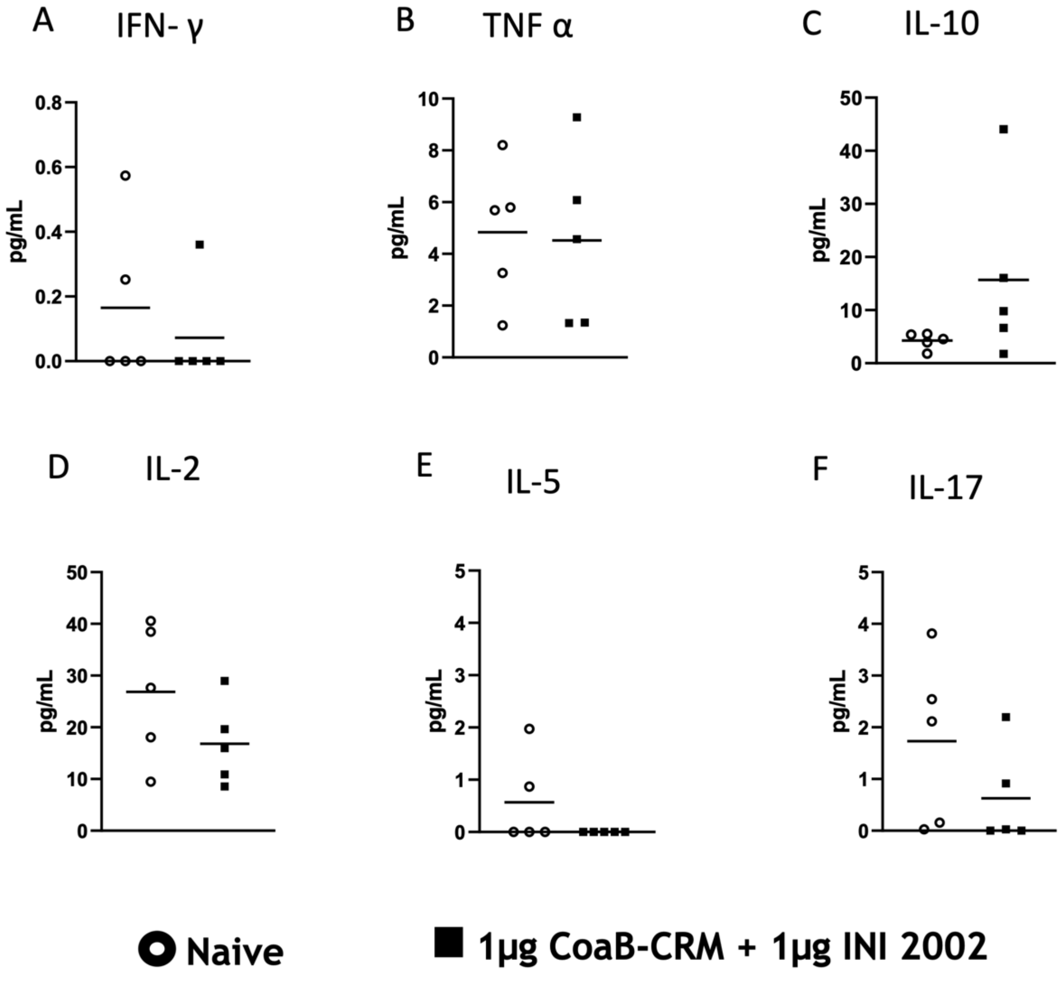

Supplemental Figure S1. INI-2002 in combination with CoaB-CRM antigen promotes cell mediated immunity to CRM. 5 days post-secondary vaccination mice were euthanized and spleens were harvested, disaggregated, and restimulated with 5 μg/mL of CRM-197. The following cytokines: IFNγ (a), TNFα (b), IL-10 (c), IL-2(d) IL-5 (e). IL-17 (f) were measured by MSD cytokine array.
